# Anticancer and Immunomodulatory Activities of a Novel Water-Soluble Derivative of Ellipticine

**DOI:** 10.3390/molecules25092130

**Published:** 2020-05-01

**Authors:** Regiane Costa de Oliveira, Gemilson Soares Pontes, Aleksandr Kostyuk, Gabriel B. Coutinho Camargo, Anamika Dhyani, Tetiana Shvydenko, Kostiantyn Shvydenko, Andriy Grafov

**Affiliations:** 1 Programa de Pós-Graduação em Hematologia, Universidade do Estado do Amazonas, Av. Djalma Batista, 3578-Flores, Manaus-AM, Brazil; regiane.costa17@gmail.com (R.C.d.O.); gemilson.pontes@inpa.gov.br (G.S.P.); CoutinhoCamargo@gmail.com (G.B.C.C.); 2 Instituto Nacional de Pesquisas da Amazônia, Av. André Araújo, 2.936-Petrópolis-Manaus-AM, Brazil; Dhyani@gmail.com; 3 Institute of Organic Chemistry, National Academy of Sciences of Ukraine, Murmanska Str. 5, 02660 Kyiv, Ukraine; a.kostyuk@yahoo.com (A.K.); shved1977@ua.fm (K.S.); 4 JSC “Farmak”, Kyrylivska str. 63, 04080 Kyiv, Ukraine; 5 Department of Chemistry, University of Helsinki, 00014 Helsinki, Finland; andriy.grafov@helsinki.fi

**Keywords:** ellipticine, 9-bromoellipticine, sodium 9-bromo-5,11-dimethyl-6H-pyrido[4,3-b]carbazole-7-sulfonate, immunomodulation, antitumor molecule

## Abstract

Cancer still remains a major public health concern around the world and the search for new potential antitumor molecules is essential for fighting the disease. This study evaluated the anticancer and immunomodulatory potential of the newly synthetized ellipticine derivate: sodium bromo-5,11-dimethyl-6H-pyrido[4,3-b]carbazole-7-sulfonate (Br-Ell-SO_3_Na). It was prepared by the chlorosulfonation of 9-bromoellipticine. The ellipticine-7-sulfonic acid itself is not soluble, but its saponification with sodium hydroxide afforded a water-soluble sodium salt. The cytotoxicity of Br-Ell-SO_3_Na was tested against cancerous (K562 cell line) and non-cancerous cells (Vero cell line and human peripheral blood mononuclear cells (PBMC)) using a Methylthiazoletetrazolium (MTT) assay. Cell cycle arrest was assessed by flow cytometry and the immunomodulatory activity was analyzed through an enzyme-linked immunosorbent assay (ELISA)_._ The results showed that the Br-Ell-SO_3_Na molecule has specific anticancer activity (IC_50_ = 35 µM) against the K562 cell line, once no cytotoxicity effect was verified against non-cancerous cells. Cell cycle analysis demonstrated that K562 cells treated with Br-Ell-SO_3_Na were arrested in the phase S. Moreover, the production of IL-6 increased and the expression of IL-8 was inhibited in the human PBMC treated with Br-Ell-SO_3_Na. The results demonstrated that Br-Ell-SO_3_Na is a promising anticancer molecule attested by its noteworthy activity against the K562 tumor cell line and immunomodulatory activity in human PBMC cells.

## 1. Introduction

Pharmaceutical companies continue to pursue antineoplastic medicines rigorously, on account of the increasing global cancer burden [1]. Despite the development of more selective therapies, treatments comprising of cytotoxic drugs are broadly used [2]. As a result, the demand for new, less toxic, and more efficient anticancer agents is of the utmost importance.

Recently, monoterpene indole alkaloid ellipticine and its derivatives have been thoroughly studied as therapeutic agents against different types of cancer [3]. Ellipticine is one of the simplest naturally occurring alkaloids with a planar structure, which has been the center of attention of many research groups because of its antitumor activity. It was first isolated from the leaves of a tropical plant *Oschrosia elliptica* by S. Goodwin et al. [4]. Ellipticine exhibits rather limited toxic side effects and a complete absence of hematological toxicity [5]. Subsequently, ellipticine was isolated from several other plants of the *Ochrosia* genus, such as *O. vieillardii*, *O. acuminate*, and *O. moorei*, as well as from *Strychnos dinkagei* (Loganiaceae family). Now, ellipticine of a plant origin represents the largest proportion of commercially available products. Different hypotheses on ellipticine action mechanisms have been proposed [6]. In the last decade, evidence of the distinct cell-cycle effects of ellipticine have come to light [7].

Particularly, ellipticine can interact with the p53 tumor suppressor protein, Akt-kinase, and c-Kit kinase, while its effect on other cellular proteins remains unclear. Thus, ellipticine exhibits a multimodal cytotoxic activity, which is not clearly specified. A biooxidation pathway was originally proposed [8], suggesting that ellipticine could serve as a substrate for peroxidases in vivo. Stiborová’s group demonstrated that ellipticine covalently binds to DNA after being enzymatically activated with cytochromes P450 or peroxidases [9]. Ellipticine derivatives such as 9-hydroxy-2-methylellipticinium acetate (NHME) had been introduced into the market, but were later withdrawn. [6] The search for other ellipticine derivatives is under way [10,11,12,13,14,15,16]. However, low water solubility of ellipticine derivatives is a crucial obstacle for the wide practical application in cancer therapies.

The aim of this study was to prepare a new water soluble ellipticine derivate, sodium 9-bromo-5,11-dimethyl-6H-pyrido [4,3-b]carbazole-7-sulfonate (Br-Ell-SO_3_Na), and investigate its antitumor and immunomodulatory potential.

## 2. Results

### 2.1. Synthesis of Sodium 9-bromo-5,11-dimethyl-6H-pyrido[4,3-b]carbazole-7-sulfonate (***2***)

**Figure 1:**
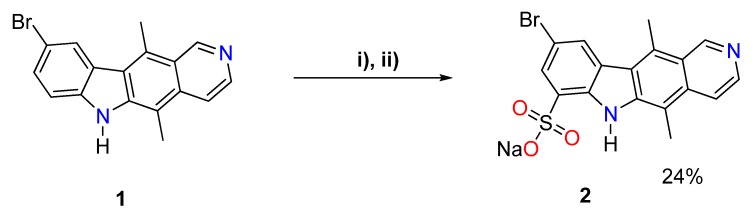
Synthesis of sodium 9-bromo-5,11-dimethyl-6H-pyrido[4,3-b]carbazole-7-sulfonate (**2**)–Br-Ell-SO_3_Na. (***i***) ClSO_3_H, 0 °C for 5 min, then r.t. for 2 h; (***ii***) chilled aqueous NaOH ~20%. The precipitate of (**2**) was washed with cool water.

9-Bromoellipticine (**1**) was prepared in gram quantities according to Cranwell and Saxton’s procedure [17], further optimized by the group. A detailed description of the synthetic procedures as well as NMR spectra are given in the Supporting Information. Sodium 9-bromo-5,11-dimethyl-6H-pyrido[4,3-b]carbazole-7-sulfonate (**2**) was obtained as shown in Figure 1, by reacting bromoellipticine **1** with an excess of chlorosulfonic acid, followed by treatment with aqueous sodium hydroxide to yield the sodium salt (Figure 1).

### 2.2. Cytotoxic Effect

**Figure 2:**
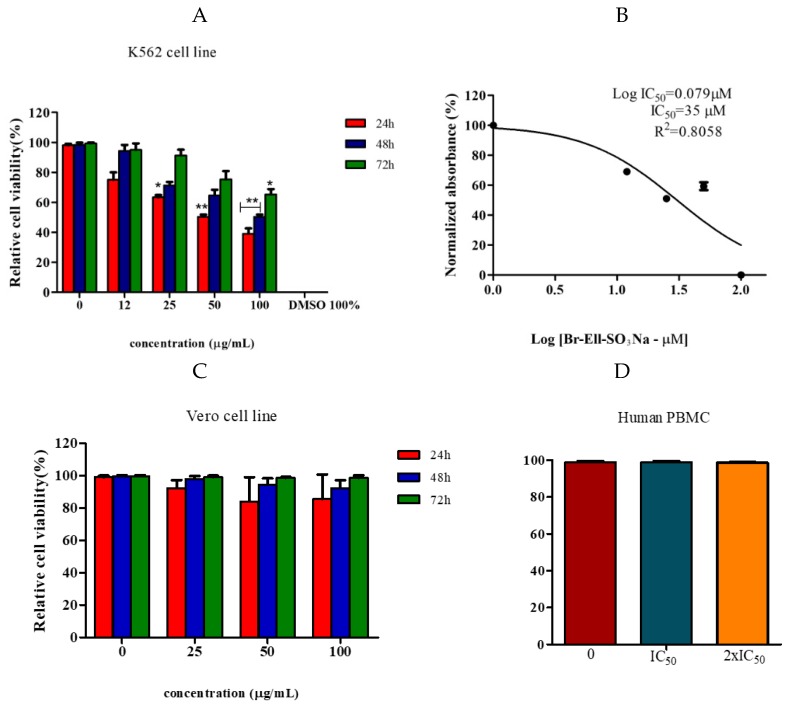
Cytotoxicity effect of Br-Ell-SO_3_Na on cancerous and non-cancerous cells. (**A**) K562 cell viability after 24–72 h treatment with different concentrations of Br-Ell-SO_3_Na (12–100 µg/mL). (**B**) IC_50_ estimation using nonlinear regression (GraphPad Prism 5 software). The absorbance values were measured at 570 nm wavelength and the mean values + SD of three experiments are displayed along with a representative IC_50_ curve. Vero (**C**) and human peripheral blood mononuclear cells (PBMC) (**D**) cell viability after 24–72 h treatment with different concentrations of Br-Ell-SO_3_Na. The Methylthiazoletetrazolium (MTT) assay was performed to estimate the cell viability/cytotoxicity. * *p* = 0.01; ** *p* = 0.001.

The compound Br-Ell-SO_3_Na exhibited anticancer activity against the leukemic cell line K562 with an IC_50_ value of 35 µM. However, the compound gradually lost its anticancer activity after 24 h treatment (Figure 2A). Thus, our findings demonstrated that the Br-Ell-SO_3_Na had effective anticancer activity against the cell line K562, which was limited to the 24 h-treatment period. Toxicity of the Br-Ell-SO_3_Na across non-cancerous cells was assessed using the Vero cell line and Human peripheral blood mononuclear cells (PBMC). Our findings showed no cytotoxicity action at the concentrations tested (Figure 2C,D), which indicates the selective cytotoxicity activity of the Br-Ell-SO_3_Na against the K562 cell line.

### 2.3. Cell Cycle Analysis

**Figure 3:**
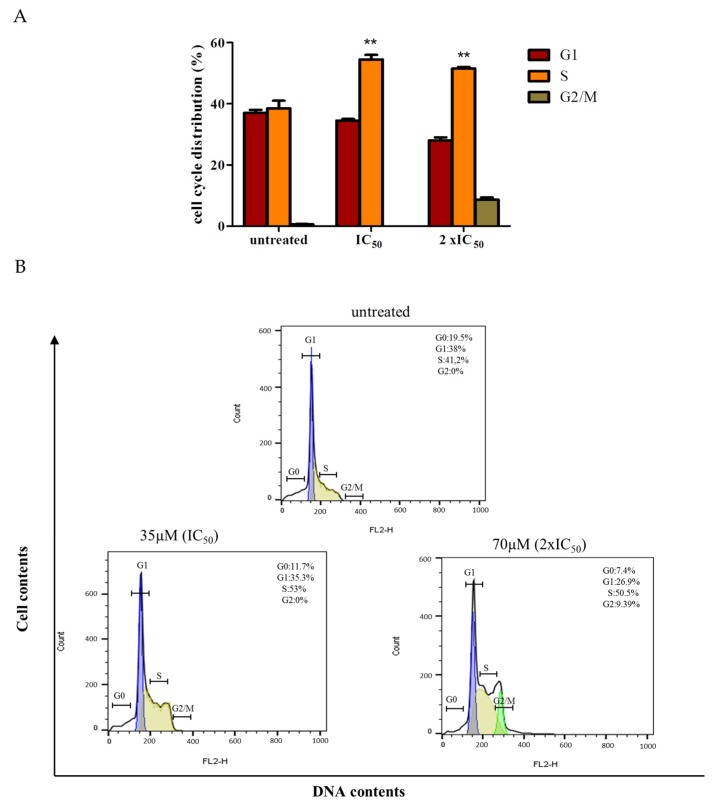
Cell cycle analysis of K562 cells treated with Br-Ell-SO_3_Na. (**A**) Analysis of the cell cycle arrest distribution in untreated (control) and treated (IC_50_ and 2 × IC_50_) K562 cells. (**B**) Representative cell cycle micrographs of K562 sensitive cells, depicting G0, G1, S and G2/M cell populations in untreated and treated K562 cells. ** *p* = 0.001.

As mentioned earlier, the K562 cells were treated for 24 h with IC_50_ (35 µM) and 2 × IC_50_ (70 µM) of Br-Ell-SO_3_Na and then the cell cycle phase was analyzed. Upon treatment with 35 µM, the S phase distribution increased significantly (53%), when compared to the control. No cells in the G2 phase were found, thus suggesting the cell cycle arrests at the S phase. This means that the Br-Ell-SO_3_Na inhibits the cell cycle progression (Figure 3). These results are concomitant with the MMT assay, where the 25 µg/mL and 50 µg/mL treatment showed a moderate antiproliferative activity after the 48 h treatment period. The cell cycle arrest remained at the *S*-phase, upon doubling the concentration to 70 µM; however, a slight increase in the G2/M phase (9.39%) was also observed.

### 2.4. Immunomodulatory Activity

**Figure 4:**
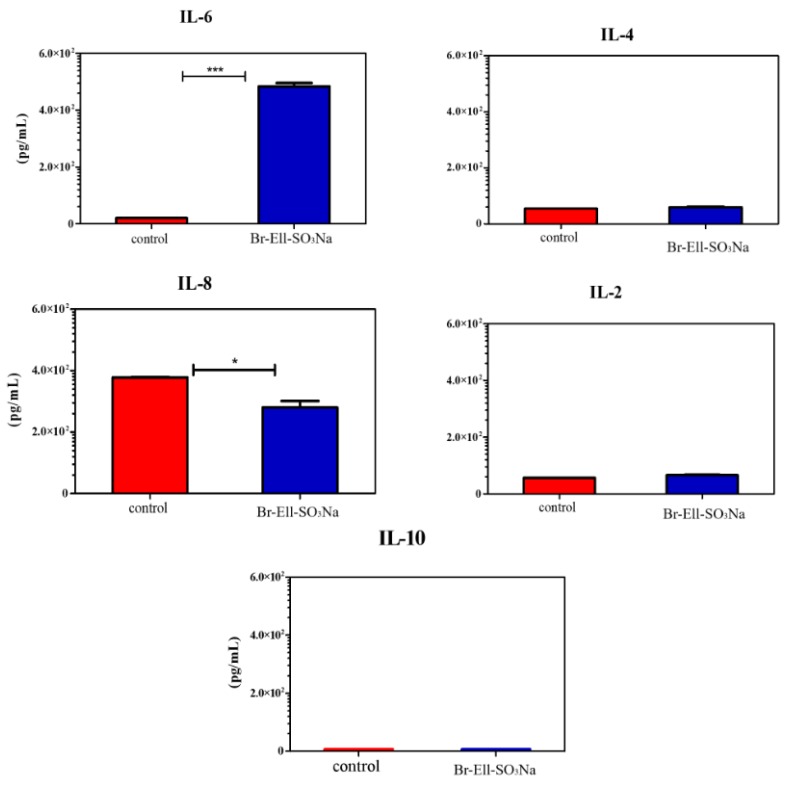
Effect of Br-Ell-SO_3_Na on modulation of the immune system. Levels of IL-2, IL-4, IL-6, IL-8, and IL-10 were assessed by ELISA in supernatant of human PBMCs treated with Br-Ell-SO_3_Na (35 µM). Controls represent the supernatant of untreated PBMCs. * *p* = 0.01; *** *p* = 0.001.

To investigate the potential of Br-Ell-SO_3_Na in modulating the immune system, the levels of IL-2, IL-4, IL-6, IL-8, and IL-10 were assessed for the K562 cell line in the supernatant of human PBMC treated with (2) at the IC_50_ = 35 µM concentration. The results demonstrated that Br-Ell-SO_3_Na stimulated the IL-6 production (*p* = 0.0001), but inhibited the expression of IL-8 (*p* = 0.02) in comparison with untreated human PBMCs used as the control (Figure 4). No immunomodulatory activity related to IL-4, IL-2, and IL-10 was observed.

## 3. Discussion

Mortality from cancer has steadily increased over time across the world, turning this non-communicable disease into the second leading cause of death globally [18]. Although substantial advances in cancer treatment have been achieved over the past decades, critical issues such as high toxicity, drug solubility and bioavailability, cost-benefit assessment, and efficacy remain the major bottlenecks in anticancer drug development.

Taking into account the wide interest in ellipticine, various synthetic routes to the main molecule and its derivatives have been developed, starting from R. Woodward’s approach [19]. Amongst a number of these available synthetic routes, the method originally proposed by Cranwell and Saxton appeared to be the most practical one [17]. The synthesis starts from appropriately substituted indoles or carbazoles. Since then, various improvements have been proposed to make it more convenient and to increase the total yield. We have also used this approach, starting from 5-bromoindole. It should be noted that the method is reproducible and was used to obtain many different substituted ellipticines. We carried out the multi-step synthesis several times in order to prepare the starting product 9-bromoellipticine in gram quantities. In addition, several improvements at different stages were proposed, to increase the total yield and to avoid column-chromatography at the isolation and purification steps (see the Supporting Information).

The low solubility of ellipticine derivatives hinders their use in medicine. As a solution, we decided to introduce an acidic group into the ellipticine scaffold. The ellipticine is susceptible to electrophilic attacks and has two electron-rich sites at the 7th and 9th positions. Only very strong electrophilic reagents can react at both positions; for example, upon bromination [20]. There are a scarce number of derivatives prepared by modification of the scaffold. The majority of those modifications relate to reactions at nitrogen atoms, electrophilic reactions at the 9th position, and modification at the 1st position. Our idea was to prepare 9-bromoellipticine, where the 9th position was blocked for an electrophilic reaction. Thus, the only position available for the electrophilic attack would be at the C7. Carbazoles are known to react with chlorosulfonic acid, hence we used an analogous procedure for the introduction of a sulfonic group into ellipticine [21]. Upon the addition of chlorosulfonic acid, 9-bromoellipticine reacted at the 7th position, giving rise to a sulfochloride derivative. The hydrolysis of the latter yielded the corresponding sulfonic acid. Since it was not soluble in water or common organic solvents, it was impossible to analyze and confirm the molecular structure of the compound. The saponification of the acid with sodium hydroxide produced the sodium sulfonate **2**. The compound is readily soluble in water and was fully characterized.

The antitumor activity of ellipticine and its derivatives have been widely demonstrated over the years [22,23,24]. However, very low solubility combined with severe side effects have been the major limitation of these compounds [22,25]. The water-soluble ellipticine derivative Br-Ell-SO_3_Na, presented by this study, inhibited the K562 cells proliferation with the IC_50_ value of 35 µM and showed no cytotoxicity to non-cancerous cells. Considering that tumorous cells stimulate their own growth by constraining inhibitory signals that control cell proliferation, it was important to verify the effect of Br-Ell-SO_3_Na on cell cycle arrest. Our findings showed that most of the cells were significantly arrested on the phase S, upon Br-Ell-SO_3_Na treatment at both 35 µM and 70 µM doses. As mentioned earlier, the anticancer potential of ellipticine and its derivatives has already been reported elsewhere, but usually those substances induce the cell arrest at the phase G2 of the cell cycle [26,27,28]. Our data demonstrated that the mechanism of action of Br-Ell-SO_3_Na on the cell cycle differs from previously reported ellipticine derivatives.

The regulation of cell proliferation is an orchestrated process that is operated by various control points at different phases of the cell cycle [29]. Any mistake at those control points may lead to the cell death. Furthermore, the physiological arrest at cell cycle check points allows a repair of cellular damage, dismissal of exogenous stress, and accessibility of nutrients [30]. However, when a negative regulation of the phase S is induced by a substance such as Br-Ell-SO_3_Na, it means that (**2**) is leaving the cell unable to duplicate its DNA by causing DNA-damage or replicative stress [29]. Indeed, intercalation into the DNA and the inhibition of a DNA topoisomerase II activity are elicited as the key mechanisms by which ellipticine employs its cytotoxicity activity [22,31]. The intercalation in DNA is believed to be facilitated by the similarity of the purine-pyrimidine base paired with the size and planar molecular structure of ellipticine [31]. The hydrophobic interaction fostered by the DNA bases and the polycyclic aromatic ring of ellipticine heightens this intercalation, which eventually results in DNA damage and cell death [32]. Although these data suggest that the Br-Ell-SO_3_Na action mechanism is related to the impairment of DNA synthesis in cancerous cells, we did not check the direct influence of Br-Ell-SO_3_Na on the intercalation of DNA or DNA topoisomerase II activity in this study.

The pathogenesis and treatment of cancer are directly interrelated with the immune system [33]. For instance, the immune-suppressive status usually induced by tumor-pathogenesis can be switched to immune-active by the immunomodulating effects of anticancer drugs, such as immune checkpoints inhibitors [34,35]. In this sense, the most promising chemical agents in the battle against cancer are the ones that can modulate the immune system through the suppression or enhancement of immune response by altering its type, scope or duration [36,37]. We assessed the immunomodulatory potential of Br-Ell-SO_3_Na, by checking the production of IL-2, IL-4, IL-6, IL-8 and IL-10 in human PBMC treated with the substance. Our results demonstrated that the Br-Ell-SO_3_Na up-regulated the expression of IL-6. Previous reports have indicated the IL-6 as a crucial player in mobilizing anti-tumor T cell immune response, and in the activation, proliferation and survival of lymphocytes during active immune responses [38,39]. Most importantly, the IL-6 signaling can also reframe the T cell immune response, converting it from a suppressive to a responsive state that is important to fight tumors [40]. On the other hand, this cytokine has been broadly linked to tumor progression and metastasis [41,42]. Thus, the role of the IL-6 trans-signaling in the context of cancer might be dependent on many factors of the tumor microenvironment, such as cell type, tumor location or severity of cancer [39].

Surprisingly, the compound Br-Ell-SO_3_Na downregulated the expression of IL-8, compared to the control. Although IL-8 and IL-6 are pro-inflammatory biomarkers that are usually stimulated together in the scenario of disease, their expression and production can be likewise differentially regulated by substances (via cAMP), as occurred in the case of Br-Ell-SO_3_Na [43]. IL-8, also known as CXCL8, is a proinflammatory CXC chemokine. The IL-8 mediates its biological activity through the binding to two G protein–coupled receptors, named CXCR1 and CXCR2 [44]. In the microenvironment of cancer cells, the IL-8 has been associated with metastasis, angiogenic response, and inducing the proliferation, survival, and migration of vascular endothelial cells [45]. The Br-Ell-SO_3_Na had no effect on the levels of IL-2, IL-4 and IL-10. The main source of IL-2 is the Th1 (cellular) immune response, whereas IL-4 and IL-10 are Th2 (humoral) cytokines [46]. The Th1/Th2 balance is critically mediated by these cytokines, which are directly involved in the inflammation-driven carcinogenesis process [47]. Our findings suggest that the immunomodulatory activity of Br-Ell-SO_3_Na is related to the balance between the stimulation and suppression of immune response in the tumor microenvironment, but not to the specific induction of Th1/Th2 immune responses. However, it is important to perform more tests to verify the influence of Br-Ell-SO_3_Na on the modulation of immune system.

Subsequently, this study demonstrated that the water-soluble Br-Ell-SO_3_Na substance synthesized by our group has anticancer and immunomodulatory potential, with no cytotoxicity activity observed against non-cancerous cells. Thus, this compound may be a good candidate to be explored as an anticancer bioactive substance in medical and pharmaceutical research.

## 4. Materials and Methods

### 4.1. General Experimental Procedures

^1^H NMR spectra were recorded with a Varian VXR-300 (299.9 MHz) spectrometer. ^13^C NMR spectra were recorded with a Bruker Avance DRX 500 (125.75 MHz) spectrometer. Chemical shifts (δ) are given in ppm downfield relative to internal tetramethylsilane (TMS) for ^1^H and ^13^C. Chromatography was performed on Gerudan SI 60 silica gel. Elemental analyses were performed at the analytical laboratory of the Institute of Organic Chemistry, National Academy of Sciences in Ukraine. The analytical grade solvents and commercially available reagents were used without further purification. Melting points were determined with an electrothermal capillary melting point apparatus.

### 4.2. Preparation of 9-bromo-5,11-dimethyl-6H-pyrido[4,3-b]carbazole (***1***)

**Figure 5:**
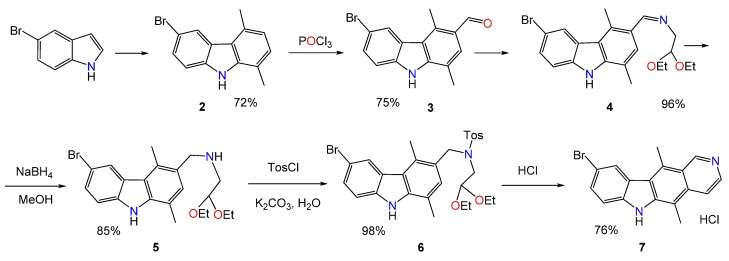
Synthesis of 9-bromoellipticine.

The compound (**1**) was prepared by the method described [17] (see Supplementary materials and Figure 5). We proposed some practical improvements. For the reaction between 5-bromoindole (**3**) and acetonylacetone, the change of ethanol to toluene, and refluxing the reagents with Dean-Stark apparatus, gave a higher yield and simplified the isolation of 6-bromo-1,4-dimethylcarbazole (**4**). At the formylation stage, we used *N*-dimethylformanilide and POCl_3_ in chlorobenzene. The aldehyde (**5**) was isolated in a 75% yield without any additional purification. The use of a slight excess of aminoacetaldehyde dimethyl acetal allowed us to isolate the corresponding imine (**6**) in an almost quantitative yield. Subsequently, the imine (**6**) was reduced to aminoacetal (**7**) by excess of sodium borohydride in methanol. The yield at that step increased to 85%. Our tosylation procedure gave an almost quantitative yield (**8**). At the final cyclization stage to 9-bromoellipticine (**1**), we used dioxane in the presence of HCl, which allowed us to avoid the chromatographic purification and to increase the cyclization yield significantly, up to 76%. Detailed information on the synthesis is given in the Supporting Information.

### 4.3. Preparation of Sodium 9-bromo-5,11-dimethyl-6H-pyrido[4,3-b]carbazole-7-sulfonate (***2***)

Chlorosulfonic acid (3 mL) was added dropwise to 9-bromoellipticine (0.1 g) at 0 °C and stirred for 5 min. The reaction mixture was continually stirred at room temperature for 2 h. The obtained brown solution was added portionwise into crashed ice, and stirred for 10 min. The yellow precipitate was filtered, washed with water (2 × 20 mL) and dried. An aqueous solution of sodium hydroxide ~20% was chilled and added carefully dropwise to the obtained 9-bromo-5,11-dimethyl-6H-pyrido[4,3-b]carbazole-7-sulfonic acid, until the pH of the product suspension reached 9. The beige precipitate was collected and washed with chilled water (2 × 1 mL). Yield 24%.

M.p. > 300 °C. ^1^H NMR (300 MHz, DMSO-d_6_): 2.73 (3 H, s, C(5) CH_3_), 3.22 (3 H, s, C(11) CH_3_), 7.76–7.77 (1H, m, C(8)H), 7.94 (1 H, d, *J* = 6 Hz, C(4)H), 8.44–8.46 (2 H, m, C(10)H and C(3)H), 9.71 (1 H, s, C(1)H), 10.01 (1 H, s, NH). ^13^C NMR (125 MHz, DMSO-d_6_): 12.1, 14.7, 109.0, 111.0, 116.5, 122.1, 122.6, 126.5, 126.9, 127.1, 129.7, 131.7, 133.4, 137.0, 139.9, 141.5, 150.4. MS (ESI): 404 (M-). Anal. Calcd for C_17_H_12_BrN_2_NaO_3_S: C, 47.79; H, 2.83; Br, 18.70; N, 6.56. Found: C, 47.89; H, 3.02; Br, 18.42; N, 6.47.

### 4.4. Cell Culture

The anticancer activity of Br-Ell-SO_3_Na was evaluated against the human leukemic cell K562 (ATCC^®^ CCL-243TM—human chronic myelogenous leukemia), since the anticancer potential of ellipticine against this cell lineage has already been reported elsewhere [48]. The selective cytotoxicity of the substance was assessed using the non-cancerous cell line Vero and human PBMCs. K562 and PBMC cells were cultured in RPMI (RPMI medium 1640/Gibco, Rockville, MD) medium supplemented with 10% heat inactivated fetal bovine serum (FBS; Gibco), 100 µg/mL Penicillin and 100 µg/mL Streptomycin (Gibco). All cells were maintained at 5% CO_2_ and 37 °C in a CO_2_ incubator. The Vero cell line were cultured in DMEM medium (with 10% FBS, penicillin-streptomycin and amphotericin B), under the same conditions described above. The cells of the K562 line were acquired from the Molecular Biology Laboratory- UNICAMP, where the cell lines had already been established and maintained. Upon written consent and after approval of the Ethical Board of the Foundation of Hematology and Hemotherapy of Amazonas (HEMOAM, approval number: 3.138.343), the PBMCs cells were obtained from the peripheral blood of healthy blood donor volunteers by density gradient centrifugation using Ficoll-hypaque (GE healthcare). Freshly isolated PBMC were used in all experiments.

### 4.5. Cytotoxicity Assay

Cytotoxicity activity of Br-Ell-SO_3_Na was determined by the Methylthiazoletetrazolium MTT assay as described elsewhere with few modifications [49]. The cell lines K562 and Vero (10^4^ cells/well) as well as PBMCs (10^5^ cells/well) were seeded into a 96-well plate and incubated for 24 h to allow the formation of the sub-confluent monolayer. After that, different concentrations of Br-Ell-SO_3_Na (12–100 µg/mL) were added into the plate wells in triplicates and incubated for 24, 48 and 72 h under the same conditions described earlier [49]. After each incubation period, 10 μL of a 5 mg/mL solution of MTT was added to the wells and incubated for 4 h. The reaction was stopped by the addition of 100 μL of 0.1 N HCl in anhydrous isopropanol. Cell growth was evaluated by measuring the absorbance at 570 nm, using an automated plate reader. Non-treated cells and 100% DMSO were used as negative and positive controls, respectively. The relative viability of cells was estimated using the following equation:

(1) $$\mathrm{Cell}\;\mathrm{viability}=\frac{A570\;\mathrm{of}\;\mathrm{treated}\;\mathrm{sample}}{A570\;\mathrm{of}\;\mathrm{untreated}\;\mathrm{sample}}\times 100\%$$

### 4.6. Cell Cycle Analysis

For determination of the cell cycle, K562 cells were seeded in a 6-well culture plate and incubated for 24 h with the compound Br-Ell-SO_3_Na at concentrations of the IC_50_ and 2 × IC_50_, determined by the cytotoxicity assay. The cells were harvested after incubation, washed with sterile phosphate buffer saline (PBS), and fixed with ice-cold 70% ethanol for 24 h at 4 °C. After washing with PBS, the cells fixed in ethanol were incubated with 100 µL of Ribonuclease inhibitor (RNAse A; 1 mg/mL) and 100 µL of propidium iodide (PI, 400 µg/mL) in an atmosphere of 5% CO_2_ at 37 °C for 30 min. The cells were then passed through a fluorescence activated cell sorter (FACS Calibur, BD Biosciences, San Jose, CA, USA) using the doublet discrimination module. Data were acquired using Cell Quest (BD Biosciences) software. The cell cycle was modelled using ModFit software (Venty Software, Topsham, ME, USA) and percentages of the cells in S, G1, and G2M phases were calculated directly by the software.

### 4.7. Cytokine Analysis

Immunomodulatory activity was assessed as described in [50] with modifications. Isolated PBMC cells were cultured in RPMI-1640 medium in a 96-well plate and incubated with different concentrations of the compound Br-Ell-SO_3_Na (IC_50_ and 2 × IC_50_) for 18 h in the CO_2_ incubator. Supernatants were collected for cytokine assays after the incubation. Concentration of the IL-2, IL-4, IL-6, IL-8 and IL-10 cytokines was evaluated by ELISA (BD Pharmingen OptEIA kits, San Diego, CA, USA), following the manufacturer’s instructions.

### 4.8. Statistical Analysis

All tests were performed in triplicate. Statistical analysis was conducted through Student’s t-test and ANOVA. A probability value of less than 0.05 was chosen as a criterion of statistical significance. The IC_50_ value was calculated by a non-linear regression.

## Supplementary Materials

The following are available online, Figure S1: ^1^H and ^13^C spectra of compound (**2**) (Br-Ell-SO_3_Na), Synthesis of bromoellipticine.doc—chemical procedures for 9-bromoellipticine.
