# Supplementary material for: Anticancer and Immunomodulatory Activities of a Novel Water-Soluble Derivative of Ellipticine
Source: Molecules. 2020 May 1;25(9):2130. doi: 10.3390/molecules25092130 (PMC7248987; doi:10.3390/molecules25092130)
Supplement: Supplementary file 1 [file molecules-25-02130-s001.zip › molecules-767012-proofed-SM/Spectra1H+13C.pdf]

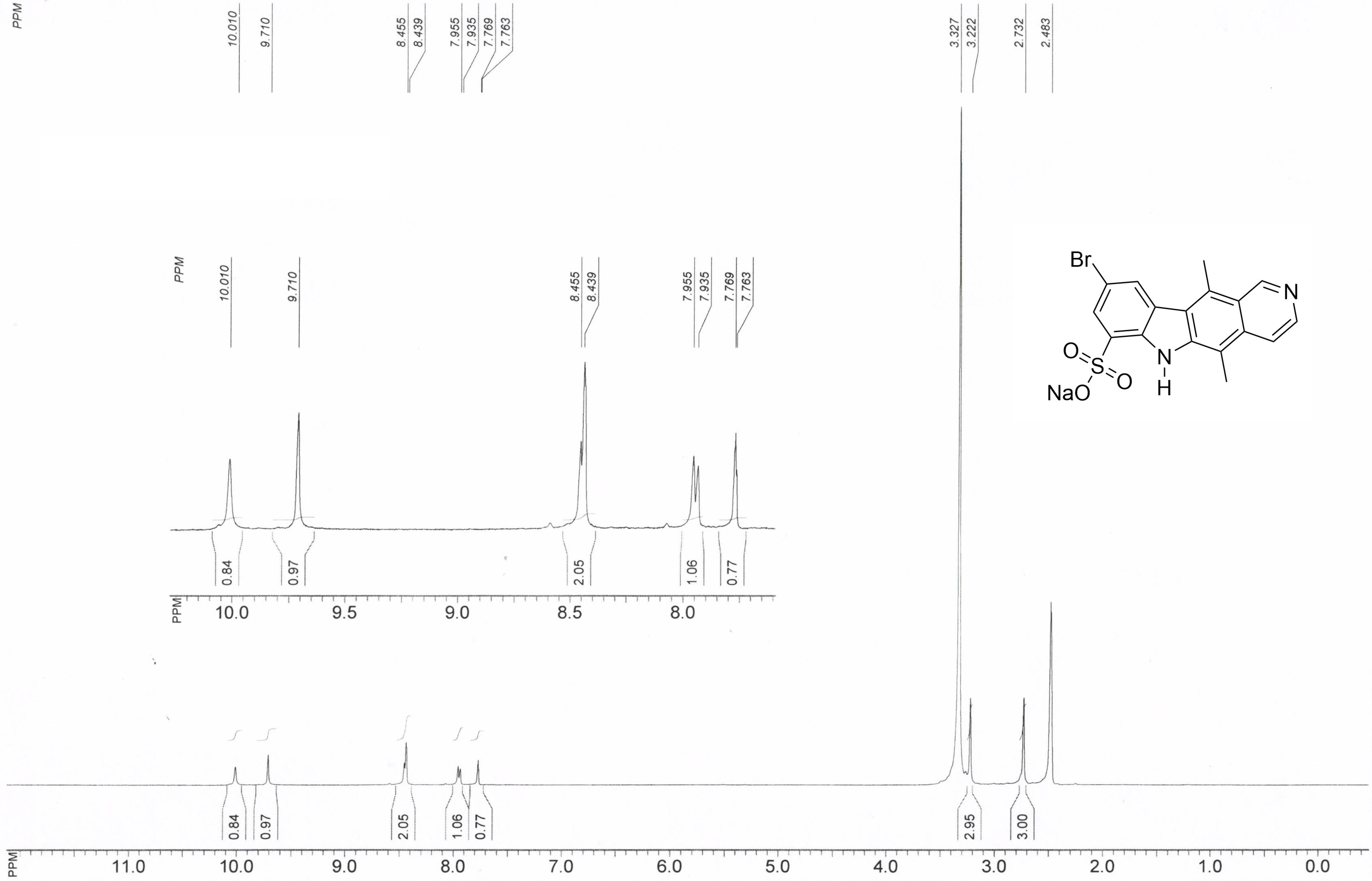

**File name: skv-12-24072018-SO3Na.fid**	Operator:	SF: 299.9530 MHz	NSC: 0	PW: 7.00 usec, RG: 20	SI: 32768
Date: 24-Jul-2018	Solvent: DMSO	SW: 4500 Hz	TE: 300 K	AQ: 2.00 sec, RD: 0.00 sec	STANDARD 1H OBSERVE

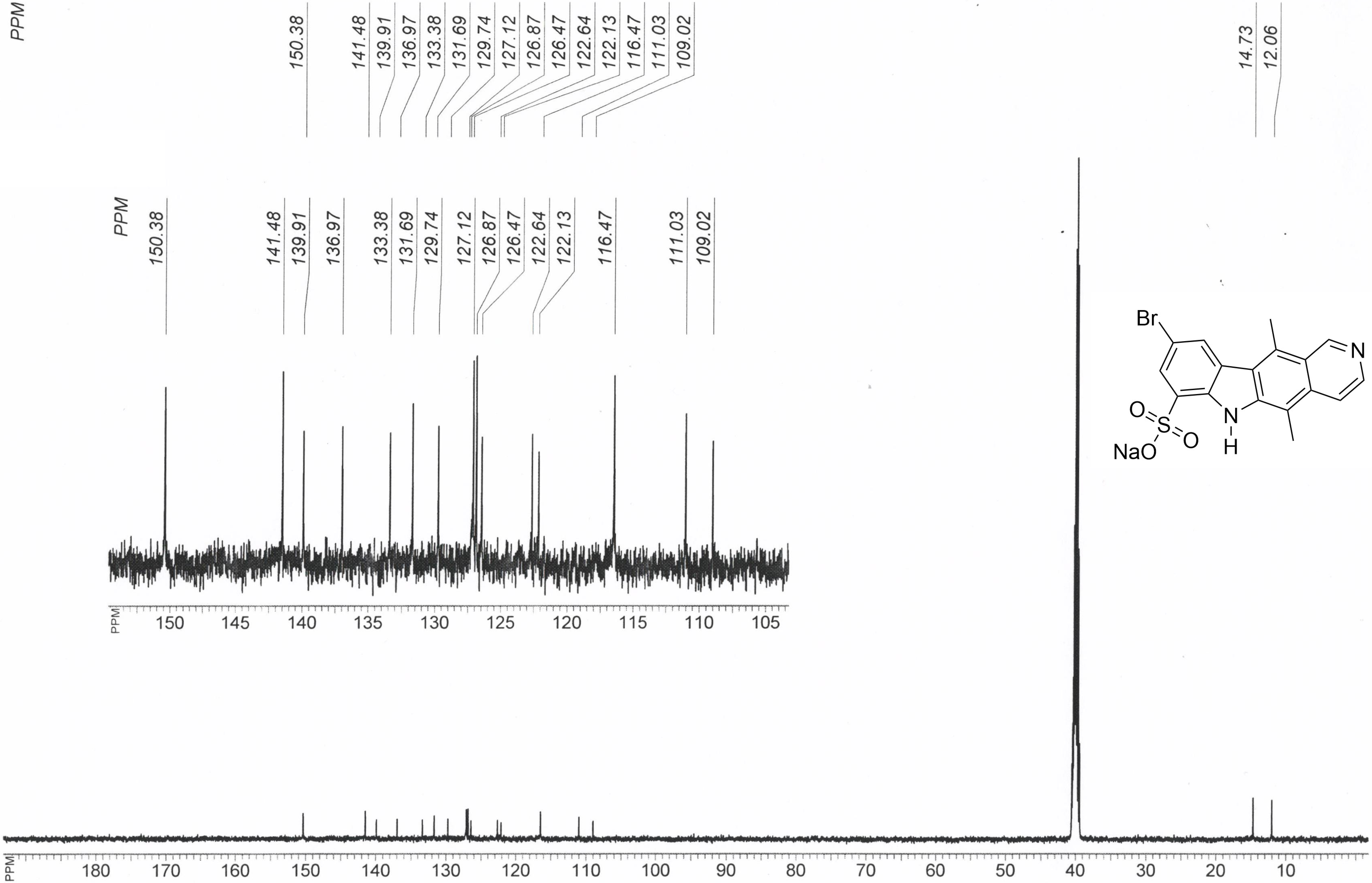

**File name: phts1117_C13**	Operator: root	SF: 125.6934 MHz	NSC: 474	PW: 0.00 usec, RG: 51200	SI: 65536
Date: 07-Jun-2018	Solvent: DMSO	SW: 32680 Hz	TE: 683 K	AQ: 1.57 sec, RD: 0.00 sec	
