# Supplementary material for: Anticancer and Immunomodulatory Activities of a Novel Water-Soluble Derivative of Ellipticine
Source: Molecules. 2020 May 1;25(9):2130. doi: 10.3390/molecules25092130 (PMC7248987; doi:10.3390/molecules25092130)
Supplement: Supplementary file 1 [file molecules-25-02130-s001.zip › molecules-767012-proofed-SM/Synthesis of bromoellipticine.pdf]

General Experimental Procedures

^1H NMR spectra were recorded with a Varian VXR-300 (299.94 MHz) spectrometer. ^{13}C NMR spectra were recorded with a Bruker Avance DRX 500 (125.75 MHz) spectrometer. Chemical shifts (δ) are given in ppm downfield relative to internal tetramethylsilane (TMS) for ^1H and ^{13}C. Chromatography was performed on Gerudan SI_{60} silica gel. Elemental analyses were performed at the analytical laboratory of the Institute of Organic Chemistry, National Academy of Sciences of Ukraine. The analytical grade solvents and commercially available reagents were used without further purification. Melting points were determined with an electrothermal capillary melting point apparatus.

Preparation of 9-bromo-5,11-dimethyl-6H-pyrido[4,3-b]carbazole

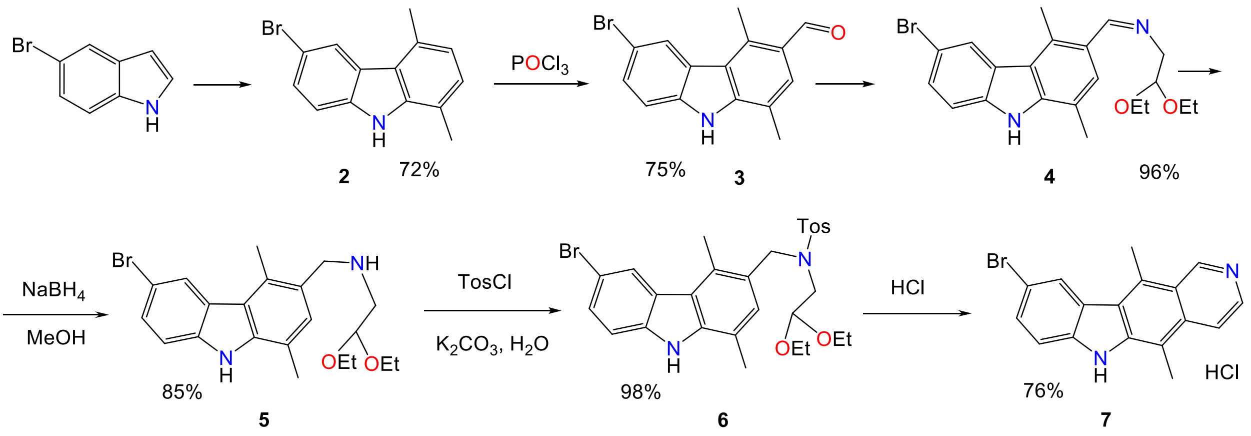

*<u>6-Bromo-1,4-dimethyl-9H-carbazole (**2**).</u>*

A single neck distilling flask 250 mL was charged with 5-bromoindole (5.3 g, 27.0 mmol), hexane-2,5-dione (3.08 g, 27.0 mmol), *p*-toluenesulfonic acid monohydrate (0.6 g, 3.1 mmol) and toluene (100 mL). It was fitted with a Dean-Stark apparatus. At first, the flask was kept at 93 ˚C for 1 h and then it was refluxed for 2.5 h collecting water. The temperature of an oil bath was maintained at 150 ˚C. The hot reaction mixture was decanted from the formed tar. The solvent was evaporated to give red powder. It was purified by flash-chromatography (eluent – hexane : ethylacetate = 1 : 1, then 100 % ethylacetate) to give (**2**) as beige crystals (5.33 g, 72%).
M.p. 136-137 °C. ^1H NMR (300 MHz, $CDCl_3$): δ 2.53 (3H, s, CH_3), 2.82 (3H, s, CH_3), 6.95 (1H, d, J = 7.3 Hz, Ar-H), 7.16 (1H, d, J = 7.3 Hz, Ar-H), 7.33 (1H, d, J = 8.5 Hz, Ar-H), 7.50 (1H, d, J = 8.5 Hz, Ar-H), 7.99 (1H, br s, NH), 8.26 (1H, s, Ar-H). Anal. Calcd for $C_{14}H_{12}BrN$: C, 61.33; H, 4.41; Br, 29.14; N, 5.11. Found: C, 61.40; H, 4.36; Br, 29.19; N, 5.18.

*<u>6-Bromo-1,4-dimethyl-9H-carbazole-3-carbaldehyde (**3**).</u>*

$POCl_3$ (2.4 mL, 25.7 mmol) was added dropwise to an ice-cold solution of *N*-dimethylformanilide (4.0 mL, 32.4 mmol) in chlorobenzene (30 mL) and then stirred at r.t. for 30 min. A solution of 6-bromo-1,4-dimethylcarbazole (5.15 g, 18.8 mmol) in chlorobenzene (30 mL) was added. The reaction mixture was heated at 105 ˚C for 4 h. The reaction mixture was cooled and an aqueous solution of sodium acetate (99 g) prepared from $CH_3COONa{\times}3H_2O$ (14 g) was added. The reaction mixture was evaporated to dryness; the residue was extracted with hot EtOAc (5 x 100 mL). The solvent was evaporated, methylene chloride (50 mL) was added,

obtained crystals were filtered, washed with methylene chloride (20 mL) to give (**3**) as beige crystals (4.25 g, 75%).
M.p. 264-265 °C. ^{1}H NMR (300 MHz, DMSO-d$_6$): δ 2.53 (3H, s, CH_3), 3.06 (3H, s, CH_3), 7.53 (1H, d, *J* = 8.4 Hz, Ar-H), 7.58 (1H, d, *J* = 8.4 Hz, Ar-H), 7.67 (1H, s, Ar-H), 8.30 (1H, s, NH), 10.35 (1H, s, Ar-H), 11.87 (1H, s, CHO). ^{13}C NMR (125 MHz, DMSO-d$_6$): 15.02, 16.99, 112.37, 113.89, 118.88, 120.79, 125.31, 125.74, 126.43, 128.56, 128.93, 136.50, 139.62, 143.09, 191.90. MS (ESI): 303 (M-H$^+$). Anal. Calcd for $C_{15}H_{12}BrNO$: C, 59.62; H, 4.00; Br, 26.44; N, 4.64. Found: C, 59.69; H, 4.07; Br, 26.38; N, 4.71.

*(6-Bromo-1,4-dimethyl-9H-carbazol-3-ylmethylene)-(2,2-dimethoxy-ethyl)-amine (**4**).*

6-Bromo-1,4-dimethyl-9H-carbazole-3-carbaldehyde (**3**) (1.5 g, 5.0 mmol) and amino-acetaldehyde dimethyl acetal (0.68 g, 6.5 mmol) was heated at 110 ˚C with stirring for 4 h. The reaction mixture was cooled, MTBE (methyl-*t*-butylether) (20 mL) was added, obtained crystals were filtered, washed with MTBE (20 mL) to give (**4**) as white crystals (1.85 g, 96%).
M.p. 183-184 °C. ^{1}H NMR (300 MHz, DMSO-d$_6$): δ 2.52 (3H, s, CH_3), 2.90 (3H, s, CH_3), 3.32 (6H, s, OCH_3), 3.73 (2H, d, *J* = 5.1 Hz, CH_2), 4.65 (1H, t, *J* = 5.1 Hz, CH), 7.53 (2H, s, Ar-H), 7.79 (1H, s, Ar-H), 8.29 (1H, s, Ar-H), 8.80 (1H, s, CH), 11.76 (1H, br s, NH). ^{13}C NMR (125 MHz, DMSO-d$_6$): 15.36, 17.13, 53.74, 63.42, 104.06, 111.60, 113.63, 118.57, 120.36, 124.98, 125.63, 125.78, 126.32, 127.80, 132.06, 139.33, 141.23, 161.78. MS (ESI): 390 (M-H$^+$). Anal. Calcd for $C_{19}H_{21}BrN_2O_2$: C, 58.62; H, 5.44; Br, 20.53; N, 7.20. Found: C, 58.58; H, 5.49; Br, 20.61; N, 7.16.

*(6-Bromo-1,4-dimethyl-9H-carbazol-3-ylmethyl)-(2,2-dimethoxy-ethyl)-amine (**5**).*

To a cooled (0 ˚C) solution of (6-bromo-1,4-dimethyl-9H-carbazol-3-ylmethylene)-(2,2-dimethoxy-ethyl)-amine (**4**) (1.35 g, 3.5 mmol) in methanol (50 mL) was added $NaBH_4$ (1.35 g, 36.5 mmol) portionwise with stirring during 15 min. The reaction mixture was kept stirring at room temperature for 1 h. The reaction mixture was evaporated to dryness, 1 M aqueous HCl was added until the solution became pH 8. The solution was extracted with ethylacetate (2 x 30 mL). The organic layer was separated, washed with brine (20 mL), dried over Na_2SO_4. The solution was evaporated to dryness to give (**5**) as white crystals (1.15 g, 85%).
M.p. 126-127 °C. ^{1}H NMR (300 MHz, DMSO-d$_6$): δ 2.53 (3H, s, CH_3), 2.66 (2H, d, *J* = 5.1 Hz, CH_2), 2.73 (3H, s, CH_3), 3.25 (6H, s, OCH_3), 3.36 (1H, br s, NH), 3.82 (2H, s, CH_2), 4.44 (1H, t, *J* = 5.1 Hz, CH), 7.16 (1H, s, Ar-H), 7.48-7.49 (2H, m, Ar-H), 8.24 (1H, s, Ar-H), 11.31 (1H, s, NH). ^{13}C NMR (125 MHz, DMSO-d$_6$): 16.03, 17.14, 50.56, 51.20, 53.70, 103.80, 110.92, 113.23, 117.43, 120.62, 124.78, 125.76, 127.48, 129.00, 129.21, 129.47, 139.27, 139.34. MS (ESI): 392 (M-H$^+$). Anal. Calcd for $C_{19}H_{23}BrN_2O_2$: C, 58.32; H, 5.92; Br, 20.42; N, 7.16. Found: C, 58.26; H, 5.88; Br, 20.48; N, 7.21.

*N-(6-bromo-1,4-dimethyl-9H-carbazol-3-ylmethyl)-N-(2,2-dimethoxy-ethyl)-4-methyl-benzenesulfonamide (**6**).*

To a mixture of 3- and 6-(2,2-diethoxyethylaminomethyl)-1,4-dimethylcarbazole (**5**) (1.0 g, 2.56 mmol) in THF (14 mL) was added water (7 mL) and then solid K_2CO_3 (0.55 g, 4.0 mmol) and *p*-toluenesulfonyl chloride (0.73 g, 3.8 mmol). The reaction mixture was stirred at room temperature for 40 h, water (50 mL) was added, the precipitate was collected by filtration and washed with 1 M aqueous HCl (20 mL), water (20 mL), saturated solution of $NaHCO_3$ (20 mL) and water (2 x 20 mL) to give (**6**) as white crystals (1.37 g, 98%).

M.p. 233-234 °C. ^{1}H NMR (300 MHz, DMSO-d_6): δ 2.39 (3H, s, CH_3), 2.45 (3H, s, CH_3), 2.71 (3H, s, CH_3), 3.05 (6H, s, OCH_3), 3.12 (2H, d, *J* = 5.1 Hz, CH_2), 4.06-4.09 (1H, m, CH), 4.53 (2H, s, CH_2), 7.04 (1H, s, Ar-H), 7.39 (2H, d, *J* = 7.9 Hz, Ar-H), 7.49 (2H, s, Ar-H), 7.73 (2H, d, *J* = 7.9 Hz, Ar-H), 8.25 (1H, s, Ar-H), 11.18 (1H, s, NH). ^{13}C NMR (125 MHz, DMSO-d_6): 15.89, 17.01, 21.40, 49.10, 51.06, 54.26, 103.18, 111.09, 113.28, 117.75, 120.59, 124.10, 124.79, 125.51, 127.54, 127.65, 129.51, 130.16, 130.30, 136.58, 139.31, 139.61, 143.58. MS (ESI): 546 (M-H^+). Anal. Calcd for $C_{26}H_{29}BrN_2O_4S$: C, 57.25; H, 5.36; Br, 14.65; N, 5.14. Found: C, 57.18; H, 5.31; Br, 14.73; N, 5.09.

9-Bromoellipticine hydrochloride (9-Bromo-5,11-dimethyl-6H-pyrido[4,3-b]carbazole hydrochloride) (7).

Compound (**6**) (1.0 g, 1.8 mmol) was suspended in dioxane (42 mL), 20% aqueous HCl (12.5 mL) was added. The reaction mixture was heated at 115 ˚C with stirring for 6.5 h. The yellow precipitate appeared in 15 min. The reaction mixture was evaporated to dryness, acetone (30 mL) was added, and the precipitate was collected by filtration and washed with acetone (2 x 20 mL). Obtained yellow crystals was washed with methanol (3 x 20 mL), combined methanol filtrates were evaporated to give (**7**) as yellow crystals (0.5 g, 76%).
M.p. >300 °C. ^{1}H NMR (300 MHz, DMSO-d_6): δ 2.75 (3H, s, CH_3), 3.14 (3H, s, CH_3), 7.53 (1H, d, *J* = 8.4 Hz, Ar-H), 7.63 (1H, d, *J* = 8.4 Hz, Ar-H), 8.11 (1H, d, *J* = 6.1 Hz, Ar-H), 8.32 (1H, s, Ar-H), 8.38 (1H, d, *J* = 6.1 Hz, Ar-H), 9.77 (1H, s, Ar-H), 12.26 (1H, s, NH). ^{13}C NMR (125 MHz, DMSO-d_6): 12.56, 15.39, 110.90, 112.92, 113.65, 119.98, 120.25, 124.25, 124.83, 126.66, 128.56, 131.29, 134.30, 134.40, 141.78, 144.45, 144.55. MS (ESI): 326 (M-H^+). Anal. Calcd for $C_{17}H_{14}BrClN_2$: C, 56.46; H, 3.90; Br, 22.09; N, 7.75. Found: C, 56.55; H, 3.83; Br, 22.15; N, 7.69.

*9-Bromoellipticine (9-Bromo-5,11-dimethyl-6H-pyrido[4,3-b]carbazole) (**8**).*

Compound (**7**) (0.3 g, 0.8 mmol) was suspended in saturated water solution of potassium carbonate (30 mL) and stirred at r.t. for 12 h. The precipitate was collected by filtration and washed with water (2 x 20 mL) to give (**8**) as greenish crystals (0.25 g, 92%).
M.p. >300 °C. ^{1}H NMR (300 MHz, DMSO-d_6): δ 2.73 (3H, s, CH_3), 3.17 (3H, s, CH_3), 7.49 (1H, d, *J* = 8.4 Hz, Ar-H), 7.64 (1H, d, *J* = 8.4 Hz, Ar-H), 7.88 (1H, d, *J* = 6.1 Hz, Ar-H), 8.38 (1H, s, Ar-H), 8.41 (1H, d, *J* = 6.1 Hz, Ar-H), 9.66 (1H, s, Ar-H), 11.51 (1H, s, NH). ^{13}C NMR (125 MHz, DMSO-d_6): 12.55, 14.97, 109.34, 111.76, 113.16, 116.93, 122.31, 123.11, 125.49, 126.46, 130.01, 130.26, 133.45, 139.84, 141.61, 142.03, 149.76. MS (ESI): 326 (M-H^+). Anal. Calcd for $C_{17}H_{13}BrN_2$: C, 62.79; H, 4.03; Br, 24.57; N, 8.61. Found: C, 62.71; H, 3.98; Br, 24.49; N, 8.66.
